# A Novel Diagnosis Method for a Hall Plates-Based Rotary Encoder with a Magnetic Concentrator

**DOI:** 10.3390/s140813980

**Published:** 2014-07-31

**Authors:** Bumin Meng, Yaonan Wang, Wei Sun, Xiaofang Yuan

**Affiliations:** The College of Electrical and Information Engineering, Hunan University, 410082 Changsha, China; E-Mails: yaonan@hnu.edu.cn (Y.W.); yuanxiaofang@hnu.edu.cn (X.Y.)

**Keywords:** rotary encoder, Hall sensor, model-based diagnosis, CORDIC algorithms, particle swarm optimization

## Abstract

In the last few years, rotary encoders based on two-dimensional complementary metal oxide semiconductors (CMOS) Hall plates with a magnetic concentrator have been developed to measure contactless absolute angle. There are various error factors influencing the measuring accuracy, which are difficult to locate after the assembly of encoder. In this paper, a model-based rapid diagnosis method is presented. Based on an analysis of the error mechanism, an error model is built to compare minimum residual angle error and to quantify the error factors. Additionally, a modified particle swarm optimization (PSO) algorithm is used to reduce the calculated amount. The simulation and experimental results show that this diagnosis method is feasible to quantify the causes of the error and to reduce iteration significantly.

## 1. Introduction

Magnetic position sensors mainly include two types. One uses a rotating permanent magnet, while others adopt a steel target wheel with teeth and slots that modulate the flux from a stationary magnet. These flux modulations can be read by semiconductors (Hall sensors or magneto resistors) [1–3]. Hall sensors are widely used to sense the rotation of moving mechanical components, they have the advantages of lower cost and higher reliability in harsh environments. Moreover, a Hall sensor-based rotary encoder has a linear response to magnetic flux density, while not having electrical hysteresis [4,5]. Hall plates placed on the periphery of a rotating magnet can be used to measure 360° absolute angle, but rotary encoders based on this principle are very sensitive to the misalignment between the sensor and magnet [2,6].

**Figure 1.**
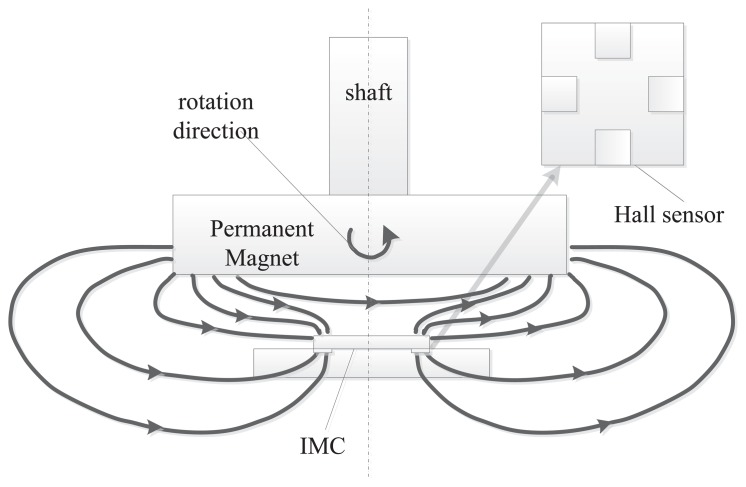
Layout and cross-section of the rotary encoder based on Hall plates with a magnetic concentrator.

To reduce the influence of the misalignment and to improve the performance, a magnetic angular sensor using Hall plates placed on the periphery of a CMOS Hall application specific integrated circuit (ASIC), which is formed an integrated magnetic concentrator (IMC), was invented [7,8]. An illustration of its layout and cross-section is shown in Figure 1. The magnetic field is parallel to a Hall sensor converted into a perpendicular field, while it passes through the IMC. The angle can be calculated by measuring the voltage of the Hall plate, which is vertically placed on the periphery of the IMC.

Different techniques, such as offset compensation, signal conditioning, spinning current Hall probe and phase compensation, have also been used to improve the performance of the rotary encoder [9–13]. Additionally, front-end calibration is performed during the final test of the sensor component, which is used to compensate for the error factors, including offset, phase and mismatch. After the assembly of the rotary encoder, the overall errors due to off-axis, tilting and magnetic error are compensated through back-end calibration.

When factors, including stray magnetic fields and the accuracy of the mechanical alignment, exceed the limit, the systematic error will get out of control. Mechanical alignment and other back-end calibration errors can result in additional offset, phase shift, amplitude change and non-linearity, which will reduce the precision of the encoder [14,15].

Using the traditional measurement methods, it is not always a simple affair to quantify each error factor after the assembly of the encoder. Model-based analytical calculation provides a way for magnetic field simulation and error analysis [15–17]. However, it is difficult to find a theoretical or experimental formula, that considers all of the factors. Yet, it still has some troubles, such as tediously long computation.

This study investigated the effects of different factors, including offset, gain mismatch, phase deviation, non-Linearity, voltage fluctuation, calculation error and other immeasurable factors. Based on the analysis of the error mechanism, an error model is built to compare the minimum residual angle error and the quantify the error factors. Moreover, a modified PSO algorithm is applied to accelerate the calculation process.

The main contribution of this paper is bridging front-end calibration and back-end calibration using the model-based method. Using this method, error sources can be quantified by parameters and front-end calibration can be performed after the assembly of the encoder. Using these parameters, the back-end compensation parameters can be changed from the discontinuity point to a continuity curve, and the small changes during the manufacturing process also can be monitored.

## 2. Error Mechanism

### 2.1. Analysis of the Error Sources

**Figure 2.**
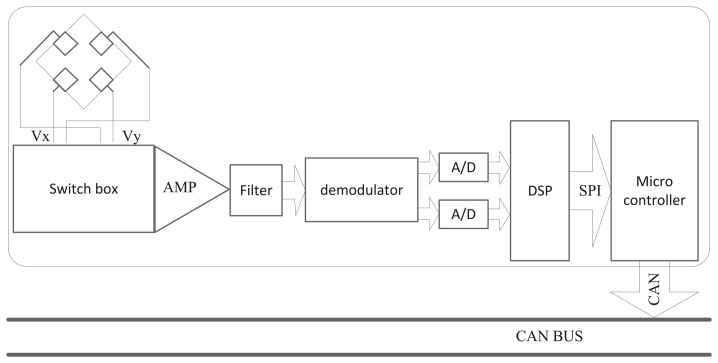
The constitution of the rotary encoder based on Hall plates with a magnetic concentrator.

The rotary encoder-based on Hall plates with a magnetic concentrator is made up of several different parts, including a switch box, a filter and amplifying circuit, a synchronous demodulator, digital signal processing (DSP), and so on. The constitution of the rotary encoder is shown in Figure 2. Both Vx and Vy are multiplexed via an adapted clock sequence for the spinning current switch boxes. After the processing of the filter, the sampling and holding are used for the demodulation. The DSP converts the voltage signal, which is amplified from the voltage on Hall plates into the angle signal. Then, the micro-controller acquires the real-time angle signal, through the serial peripheral interface (SPI). The encoded rotation angle is finally sent out by the controller area network (CAN) after compensating.

The performance of the rotary encoder depends on several factors, such as the offset, misalignment, gain mismatch, non-linearities, noise, quantization error, calculation error, and so on [12–14]. Special technology is necessary to limit the offset and ensure signal consistency. Just as with the signal processing technology shown in Figure 2, both axes are multiplexed via an adapted clock sequence for the spinning current switch boxes. Moreover, quadrature spinning and a chopper stabilized amplifier are effective ways to limit the offset. Of course, the influence of these cannot be eliminated completely [9,10].

Misalignment refers to two perpendicular Hall plates not being aligned and the magnetic field not being aligned with the Hall plates. However, in fact, the misalignment does not come from the CMOS process, because the machining precision of CMOS is up to 0.1*μ*m. The source of misalignment is the IMC, which is attached on top of the die at the end of the process line: this IMC is shifted against the die, due to the placement tolerances. Moreover, the shape of the IMC should ideally be a flat disk, but in reality, its perimeter has a poor quality, due to process reasons (rough surface with small mouse-bites, *etc.*). This leads to the orthogonal errors of both axes [18].

The process imperfections, like mask misalignment and etching tolerances, lead to mismatch in sensitivity and orthogonality between measurement axes [11,19].

Non-linearities (NL) occur with the variations of the magnetic field, which contributes to the angular measurement distortions. The magnetic NL is about 0.5 mT for a magnetic field up to 80 mT. Absolute non-linearity (ANL) is defined as the deviation from the best linearities with a unitary slope. For every sample, an ANL of the second harmonic is observed; the sources of this type of ANL will gain the mismatch and non-orthogonality of the sensor axes [9,12,14].

Analogous to digital sampling, which also brings error, the influence can be reduced through improving the sampling accuracy. Meanwhile, the calculation of the arctangent is the possible error source. The CORDIC algorithm is a general method to calculate the arctangent, whose influence is foreseeable.

Some factors, including the mechanical deviation, are easy to understand, while others are not obvious. Due to the measurement limitations, their influences cannot be quantized, but their influences can still be expected to follow the formula given below [18].

(1) $$\alpha^{'}=\text{arctan}(\frac{\text{Vy}+\text{Ay}\cos(\alpha -90^{0}+\beta )}{\text{Vx}+\text{Ax}\cos(\alpha )})$$

where:

– *α* is the angle measured by the rotary encoder, while *α* is the standard angle.
– Ax and Ay are the magnetic sensitivity along the *x*- and *y*-axes, respectively;
– Vx and Vy are the offsets of the *x*- and *y*-axes, respectively.
– *β* is the orthogonality correction angle (phase error).

Meanwhile, the influences of temperature, abrasion and the errors caused by other devices also follow the general error mechanism, but showing different characteristics in specific conditions.

### 2.2. Influence of Error Factors

#### 2.2.1. Offset Error

**Figure 3.**
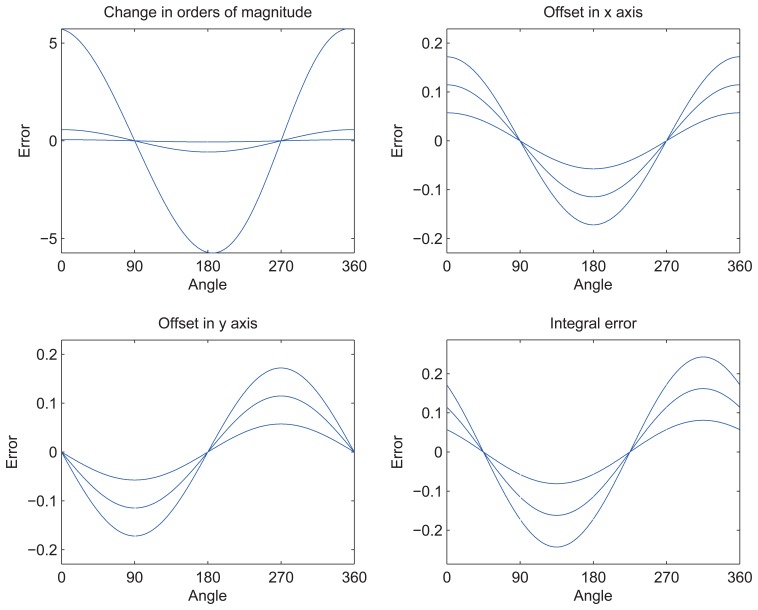
The angle error curves in different offset conditions.

Offset voltage is often small and related to temperature. The relative angle error curve in different offset conditions is shown in Figure 3.

Subgraph 1 shows the error curves when the offset of the axis changes in orders of magnitude. Subgraphs 2 and 3 show the error curves when the offset changes in the *x*-axis and *y*-axis (offset of 1 mv, 2 mv and 3 mv). Integral error shows the trend of error when the offset changes in both the *x*- and *y*-axes.

As the figure shows, offset and error change in the same order of magnitude. Degree is the unit of angle error, while volt is the unit of the corresponding offset.

#### 2.2.2. Gain Mismatch

Commonly, the mismatch exists in a unit of one in a thousand. After gain compensation using the previously measured magnetic values, the absolute error can experimental be reduced by half.

**Figure 4.**
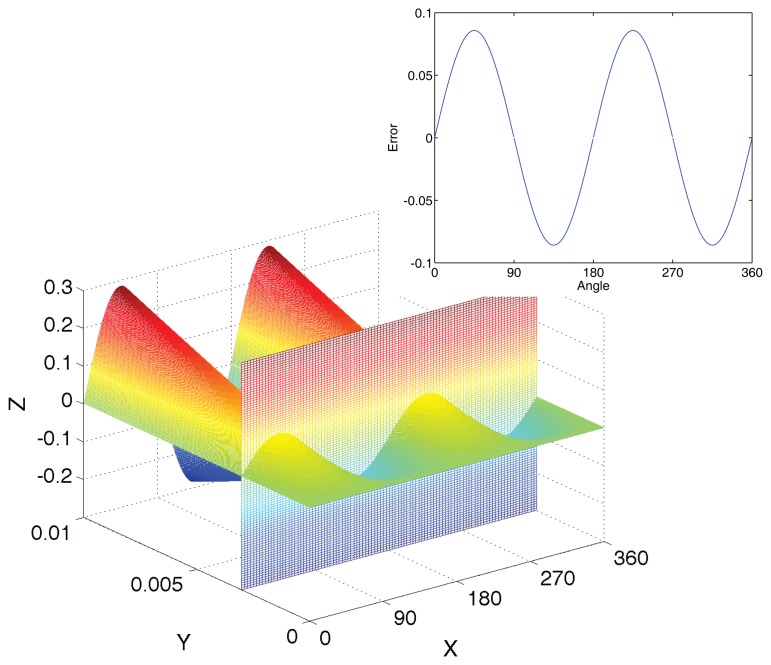
The influence of gain mismatch. The x-axis is the angle from 0° to 360°. The *y*-axis is the percentage of mismatch from 0% to 1%. The *x*-axis is the relative error.

As shown in Figure 4, the error caused by gain mismatch presents two cycle changes in 360°, and it is directly proportional to the percentage of mismatch.

#### 2.2.3. Phase Error

**Figure 5.**
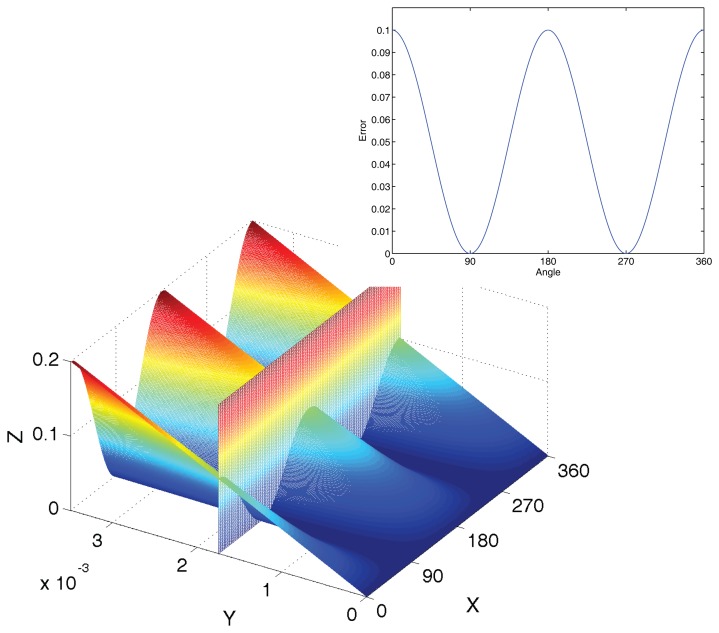
The error curve of phase deviation. The *x*-axis is the angle from 0° to 360°. The *y*-axis is the deviation of phase. The *x*-axis is the error.

Misalignment and hysteresis appear in the form of the phase error, a curve of which is shown in Figure 5. Usually, phase deviation caused by hysteresis is below 0.05°, while it can be up to 0.1° or even above, considering misalignment and other effects.

#### 2.2.4. Non-Linearity

**Figure 6.**
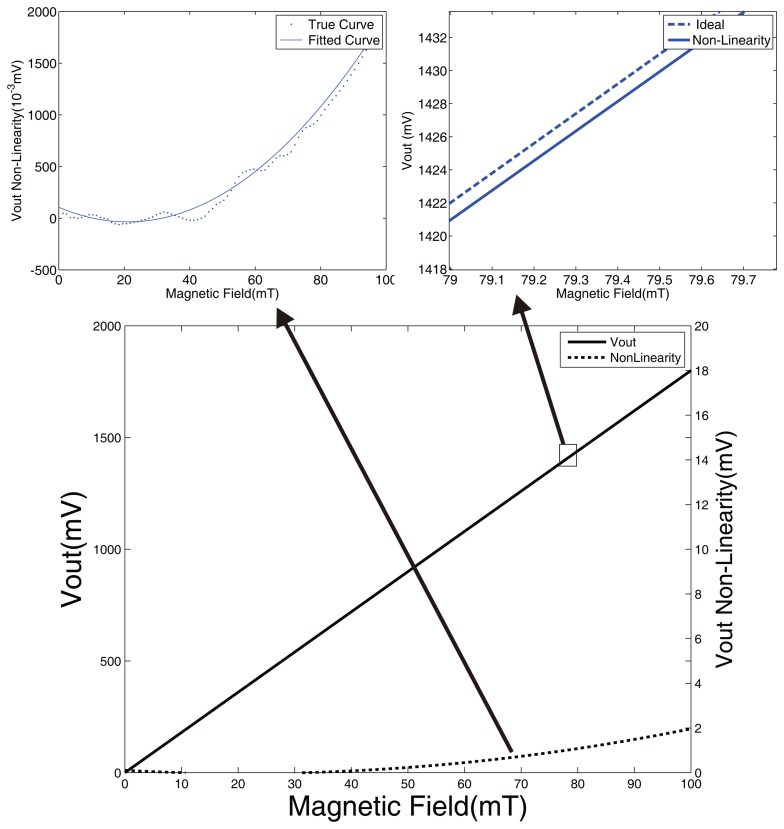
(**Upper left**) In the subgraph, the non-linearity relationship of the magnetic field intensity and voltage is represented. The fitting curve is obtained from the real curve through the polynomial fitting method; (**Upper right**) The subgraph shows the relationship of the magnetic field intensity and the output voltage.

Normally, the non-linearity of the signal is negligible. In the situation of magnetic saturation (the applied field on the IMC location is greater than the saturation value) or low magnetic field, the non-linearity is not ignorable. The relationship between magnetic field intensity and output voltage is shown in Figure 6. The election and installation of a permanent magnetic material is essential for avoiding non-linearity.

**Figure 7.**
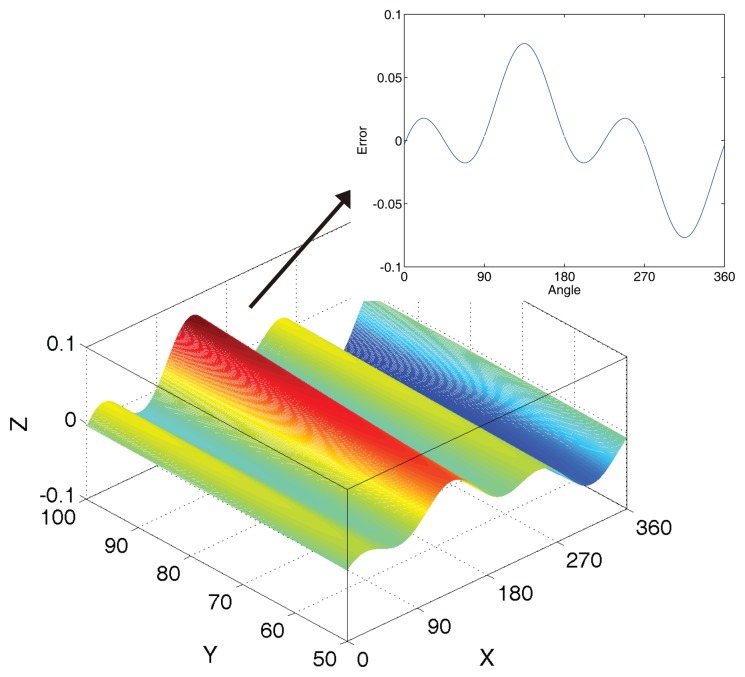
The angle error curve in non-linearity. The *x*-axis is the angle from 0° to 360°. The *y*-axis is the magnetic field intensity from 0 mT to 100 mT. The *z*-axis is the error.

The polynomial parameters of the non-linearity curve are concerned with the properties and the size of permanent magnetic materials. A formula of polynomial fitting is quadratic polynomial, as follows.

(2) $$\text{F}=\text{K}1*{\text{magx}}^{2}+\text{K}2*\text{magx}+\text{K}3$$

when K1, K2, K3 are polynomial parameters of non-linear equations from magnetic fields. magx is the maximum magnetic field intensity measured in the Hall plate. Of course, the analysis method of the magnetic field is also a way to get a fitting curve [16]. The angle error curve of non-linearity is shown in Figure 7.

#### 2.2.5. Voltage Fluctuation

Voltage fluctuation is inevitable. The volatility of the reference voltage directly leads to the sampling errors. Error *ω* related to the quantization noise of the ADC can be expressed as the following formula.

(3) $$\omega =\frac{x}{2^{\text{m}}}\Delta \text{D}$$

**Figure 8.**
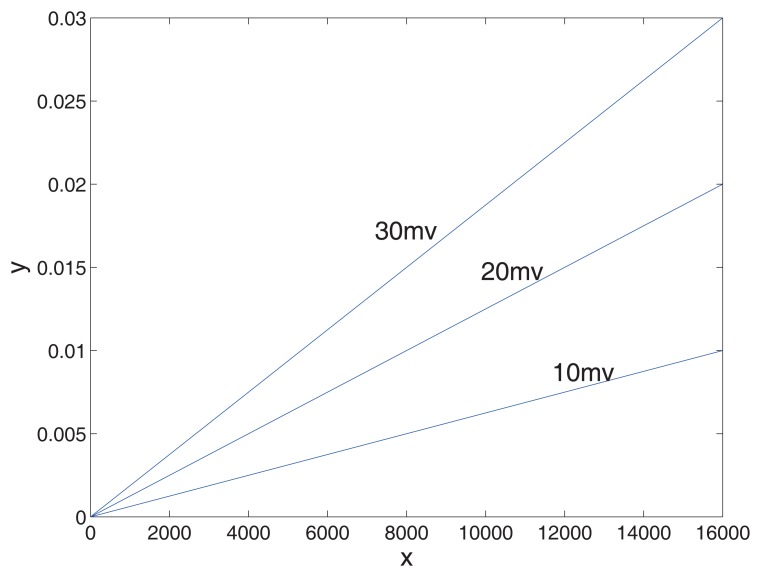
The error in different voltage fluctuations. The *x*-axis is the quantitative values of the sampling voltage. The *y*-axis is the sampling error.

ΔD is the reference value and *m* is the sampling accuracy of the ADC. In 14-bit accuracy, quantitative values of sampling voltage *x* are from zero to 16,383. The sampling error is caused by voltage fluctuation, as presented as Figure 8.

The error increases with the voltage amplitude. Essentially, the voltage fluctuation is the value of the reference voltage offset from the ideal. Usually, this inconsistency is caused by the instability of external input voltage [20,21].

#### 2.2.6. Calculation Error

Calculation of the arctangent is also a source of error. The table-lookup algorithm has the features of being fast and simple. However, it needs a large storage space, and computing precision cannot be well guaranteed [22].

Another effective calculation method is the CORDIC algorithm. The CORDIC algorithm is an iterative technique to compute several trigonometric and hyperbolic functions by using additions and shifts [23,24].

**Figure 9.**
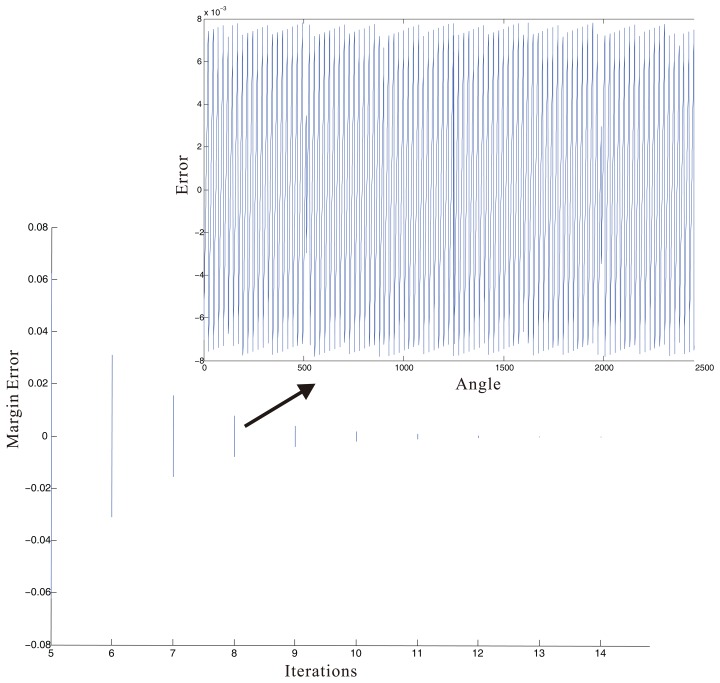
The error in different iterations. The range of the envelope curve decreases with the increase of the iterations. In the CORDIC algorithm, all angles originate from the angle range of 0° to 90°. The subgraph displays the error through eight iterations.

Errors in the CORDIC algorithm are mainly of two types: (1) the angle approximation error, which originates from the quantization of the rotation angle represented by a linear combination of finite numbers of elementary angles; (2) the finite word length of the data path resulting in the rounding/truncation of the output, which increases cumulatively through the successive iterations of micro-rotations [25]. The calculation error of a general pipelined CORDIC architecture is shown in Figure 9. Modified CORDIC algorithms can reduce the iteration time, but the changes of the error margin are similar.

### 2.3. Modeling the Errors

The influence of factors can be revealed using a single factor to calculate the error. The influence of the single factors, which approaches, reality, is presented in Table 1.

Obviously, the magnitude of error is mainly affected by the offset, gain mismatch, phase deviation, non-linearity and voltage fluctuation. Meanwhile, quantization, calculation and other factors have an impact on the fluctuation range of the error. The influences of quantization and calculation are foreseeable. Although the influences of other factors are immeasurable, they have only a limited impact. With comprehensive consideration of the Equations (1) and (2), the model to diagnose the error can be described as follows [17,26].

(4) $${\text{P}}_{\text{nk}}=\text{arctan}\left(\frac{{\text{Vy}}_{\text{k}}+{\text{Ay}}_{\text{k}}*\text{F}(\alpha_{\text{n}}-\text{pi}/2+\beta_{\text{k}})}{{\text{Vx}}_{\text{k}}+{\text{Ax}}_{\text{k}}*\text{F}(\alpha_{\text{n}})}\right)-\alpha_{\text{n}}$$

where:

– P_nk_ is the value of *α*′ −*α* when angle numbers are n and the parameter number is k.
– Vy_k_, Ay_k_, Vx_k_, Ax_k_, *β*_k_ is parameters value of number k.
– *α*_n_ is *α* with the number of n in the scope of the whole cycle.

F (*α*_n_) is defined as the formula below.

(5) $$\text{F}(\alpha_{\text{n}})=\text{K}1*{{\text{magx}}_{\text{k}}}^{2}*\cos2(\alpha_{\text{n}})+\text{K}2*{\text{magx}}_{\text{k}}*\cos(\alpha_{\text{n}})+\text{K}3$$

Now, we have a clear understanding of error factors. Additionally, there are a couple of parameters values to present the error of the encoder measurement results. Using these parameters, the error parameters of key factors can be quantified; where we still see the problem that a single error factor is immeasurable and there exist interactions among different factors; which leads us to introduce the model-based diagnosis method.

It works like this: For all of the variables, a couple of values are assumed within the possible range, and then the calculating of the results of these assumed values are compared with the actual results. By comparing the minimum residual angle error, the variables most consistent with the practice ones can be obtained. In the below formula, *R_k_* is the minimum residual angle error of a couple of values in six dimensions.

(6) $${\text{R}}_{\text{k}}=\underset{\text{k}}{\text{Min}}\left(\sum_{\text{n}}{\text{P}}_{\text{nk}}\right);{\text{Vy}}_{\text{k}},{\text{Ay}}_{\text{k}},{\text{Vx}}_{\text{k}},{\text{Ax}}_{\text{k}},\beta_{\text{k}},{\text{magx}}_{\text{k}}\in \prod \text{k}$$

The range of fluctuation is used to choose the optimal solution, as there might be more than one result.

The fluctuation can be scaled by:

(7) $$\Delta \text{E}=\sigma_{\text{rms}}+\omega +\delta +\max(\alpha_{\text{cordic}}\left(\text{iterations}\right)-\alpha );\alpha \in \left(0,2\pi \right)$$

where:

– ΔE is the fluctuation range of *α*′ −*α*.
– *σ_rms_* is the quantization error.
– *ω* is the error caused by the voltage fluctuation.
– *δ* is immeasurable error.

If the fluctuation goes beyond the range, this set of solutions should be removed.

## 3. Structure of the Diagnostic System

**Figure 10.**
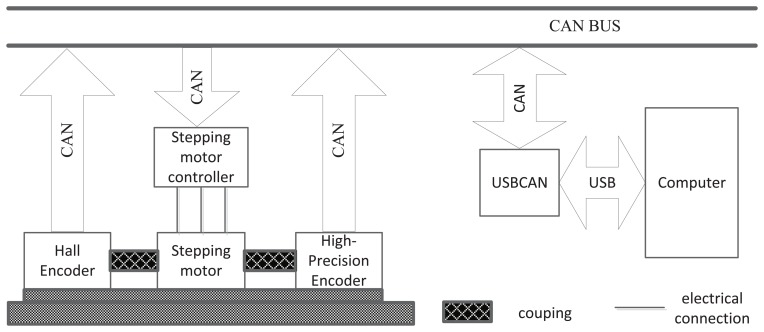
The structure of the diagnostic system.

To realize the diagnostics, a diagnostic system is presented, the structure of which is shown in Figure 10.

**Figure 11.**
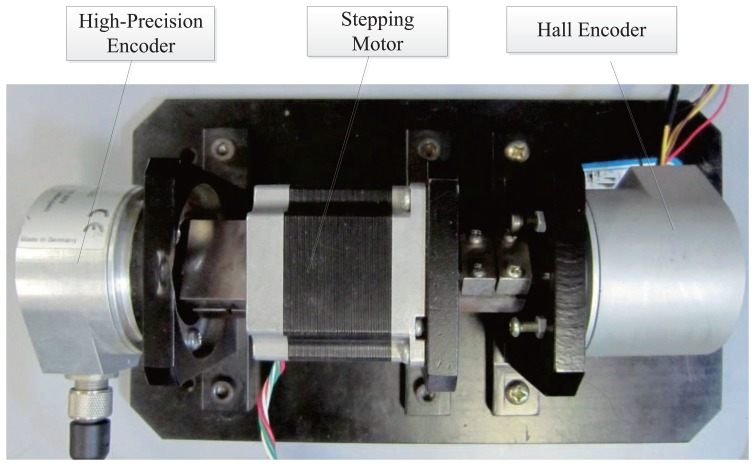
The diagnostic platform for the rotary encoder.

In this system, a high-precision encoder and a Hall encoder are used to measure the angle of a shaft while a computer controls the whole system. A USBCAN device is used to work as a router between CAN and Universal Serial Bus (USB). The computer-controlled diagnostics system works under a prescribed sequence, through receiving data and sending commands. Firstly, the stepping motor controller drives the shaft to special angle by receiving command from computer. After shaft stopped in a special angle; the computer receives the measurement results from the high-precision encoder and the Hall encoder. Through comparing two measurement results, the error of a specific angle is obtained. In the same way, the error of the whole circle can be obtained. A real picture of diagnostic platform is shown in Figure 11.

## 4. Rapid Diagnosis Method Using PSO

### 4.1. The Process of the Rapid Diagnosis Method

Through a comparison with a high-precision standard rotary encoder, the Hall encoder's error in the whole circle can be obtained. Pretreatment and preliminary judgment can be used to reject manifest errors and provide reliable data. After that, the error factors are quantified through comparing the minimum residual angle error. Using an ordinary method, it is necessary to make a calculation for every step in the range of each parameter.

The PSO algorithm has supplied an effective method for the fitting of continuous non-linear functions [27]. So as to improve efficiency, the PSO algorithm is applied to compare the minimum residual angle error and to quantify the error factors. However, there are two problems: the minimum *P n* is not always best, because of the influence of noises. Furthermore, convergence is uncertain under a certain number of iterations. To improve the applicability of the algorithm, the procedure of the modified PSO is as follows.

The variables Vx, Ax, Vy, Ay, Px and Magx make up a six-dimensional search space, and a set of variables is considered as a particle; then, the N particles constitute a group. The i-th particle position can be represented as vector Z_i_= (*X_i_*_1_, … , *X_i_*_6_). The particle speed V_i_= (*V_i_*_1_, …, *V_i_*_6_) is defined as the moving distance in each iteration. The optimal location for whole particles is the global best position P_gj_ = (*P*_g1_, … , *P*_g6_). The optimal location for the current particle swarm is the individual best position P_ij_ = (*P_i_*_1_, … , *P_i_*_6_). *R_k_* is an objective function. A particle updates its velocity and position by tracking the individual best position (this corresponds to the BestFiterror) and global best position (this corresponds to the Meanfiterror). In each iteration, the particles update the speed and position according to the following formula.

(8) $${\text{V}}_{\text{ij}}\left(\text{k}+1\right)=\omega {\text{V}}_{\text{ij}}(\text{k})+{\text{c}}_{1}{\text{r}}_{1}({\text{P}}_{\text{ij}}(\text{k})-{\text{X}}_{\text{ij}}(\text{k}))+{\text{c}}_{2}{\text{r}}_{2}({\text{P}}_{\text{gj}}(\text{k})-{\text{X}}_{\text{ij}}(\text{k}))$$

(9) $${\text{X}}_{\text{ij}}\left(\text{k}+1\right)={\text{X}}_{\text{ij}}+{\text{V}}_{\text{ij}}\left(\text{k}+1\right),\text{i}=1,2,\ldots ,\text{N},\text{j}=1,2,3,4,5,6$$

where:

– c_1_, c_2_ are accelerating constants, also known as the acceleration factor, which make the particles have a self ability to summarize and learn from other excellent individuals in groups.
– r_1_, r_2_ are random numbers between zero and one.
– i, j denote the label of the particle and the dimensions.
– V, X are the velocity and position. The velocity is multiples of steps.
– P_ij_, P_gj_ are the individual best position and global best position.
– k is the number of iterations.
– *ω* is the inertia weight, which is defined as follows.

(10) $$\omega (\text{iter})=\frac{\left({\text{Iter}}_{\max}-\text{iter})(\omega_{\max}-\omega_{\min}\right)}{{\text{Iter}}_{\max}}+\omega_{\min}$$

The inertia weight value of every particle is fixed according to its iterations to keep the balance between the local and the global searching abilities [28,29]. Additionally, *P_nk_* is calculated for every picked particle, and a particle is discarded if Equation (11) is satisfied. Comparing with standard PSO, modified PSO has better convergence speed, because of the inertia weight in Equation (10) and the condition in Equation (11).

(11) $$\text{abs}(P_{n\text{k}}-\alpha^{'}+\alpha )>\Delta \text{E}$$

**Figure 12.**
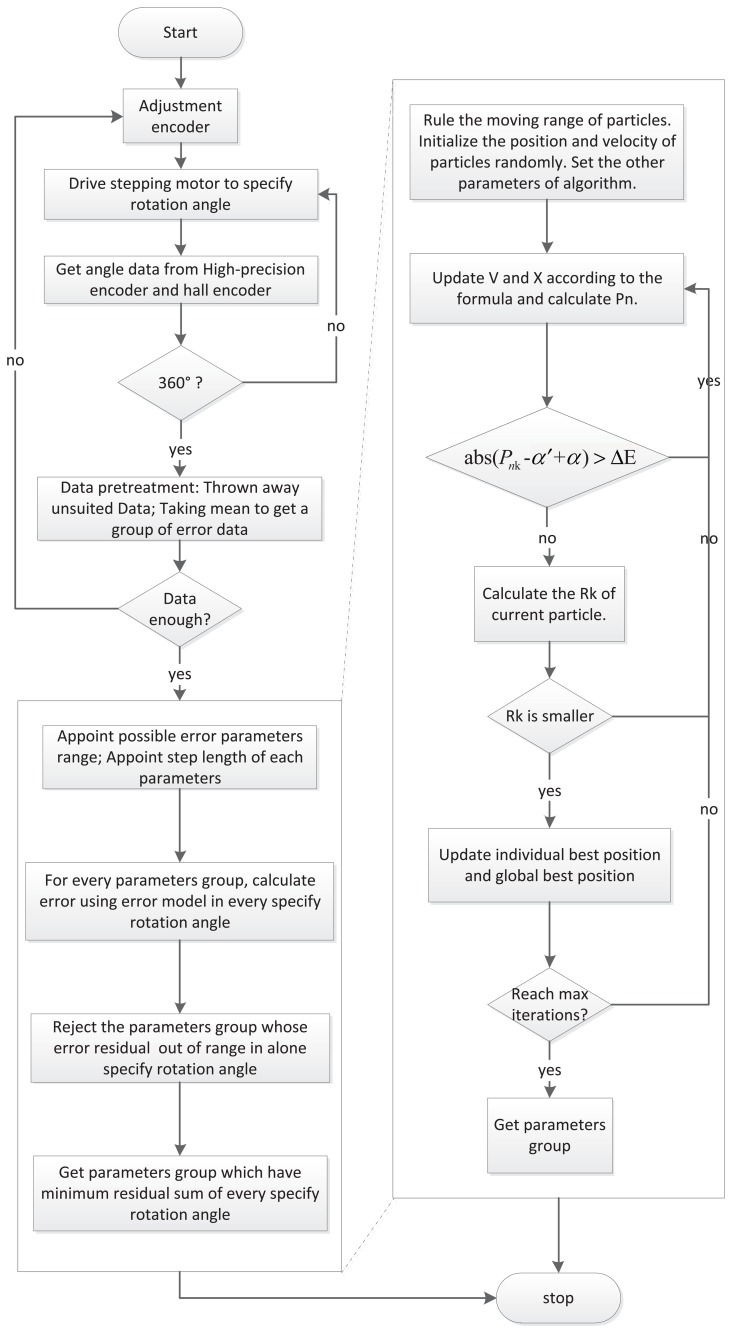
The flow chart of the rapid diagnosis method. (**Left**) The ordinary method; (**right**) the method using particle swarm optimization (PSO).

If current *R_k_* is smaller than the previous one, update the individual best position and global best position. Through finding the particle with the smallest *R_k_*, a set of the most suitable parameters can be found. The flow chart of the diagnosis method is shown in Figure 12. A rapid diagnosis method can significantly reduce the amount of calculation, as we should not have to calculate every parameter.

### 4.2. Algorithm Testing

**Figure 13.**
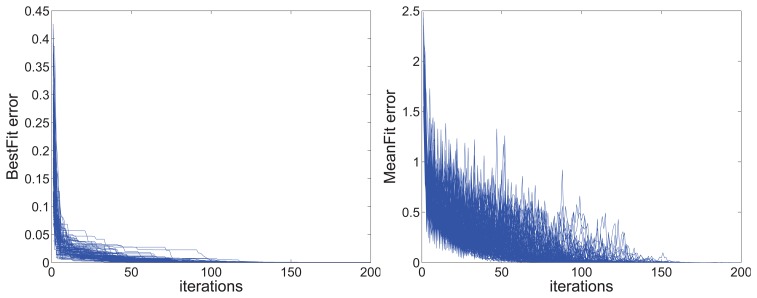
The convergence of error in the individual best position (BestFiterror) and global best position (Meanfiterror).

This algorithm must ensure that the iteration is stably convergent. To demonstrate its feasibility, the random data are used to test the convergence of the algorithm. One hundred cases are used to test the average iteration number and the rate of convergence, the results are shown in Figure 13. Just as the figure shows, the proposed method works well in different cases. It is obvious that the convergence of error in the individual best position and the global best position can meet the requirement with less than 200-times the iterations.

Take the 100 particles group, for example; a 200-times iteration means 20,000 particles are calculated. Compared to the calculated amount of directly calculating the iteration, the proposed approach has the advantage of fast speed and high feasibility.

**Figure 14.**
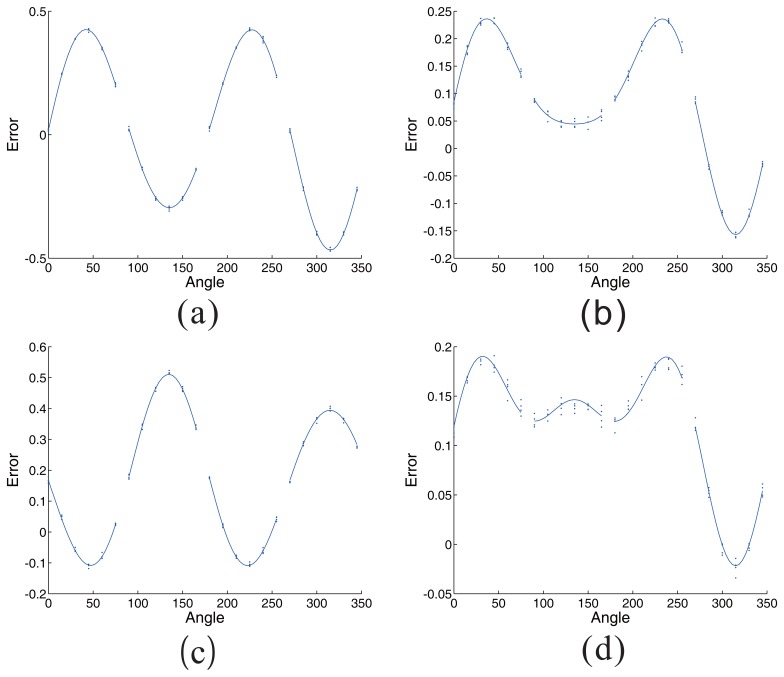
The performance of the error diagnosis method in different cases. The situations (**a**), (**b**), (**c**), (**d**) are used to acquire error parameters. The discrete points are four groups measuring errors in the same situation, while the curves are drawn using the output error parameters.

For the sensors working in different environment, there are many shapes of error. As shown in Table 2, the variables related to factors are variable in a range. The output error parameters in different situations (a, b, c, d) are demonstrated in the table. The different error shapes, related to the output error parameters, are respectively shown in Figure 14. The situations (a, b, c, d) in Table 2 are corresponding to the (a), (b), (c), (d) in Figure 14. Thereinto, the discrete points are four groups measuring errors in the same situation, while the curves are drawn using the output error parameters.

**Figure 15.**
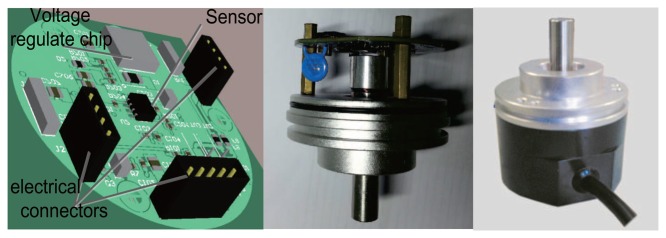
The printed circuit board (PCB) real product photos.

**Figure 16.**
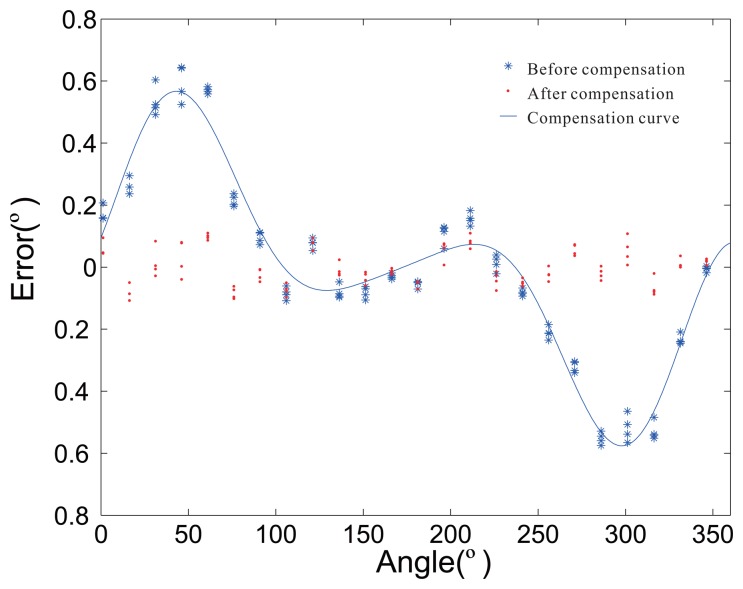
The error before and after the compensation.

Although there is not strictly a one-to-one relationship to the real situation, the parameters can reflect the changing trend of the working condition. Using the parameters, small changes during the manufacturing process can be monitored. Additional, the back-end compensation parameters can be changed from the discontinuity point to the continuity curve. The printed circuit board (PCB) and real product photos are presented in Figure 15. The error distribution before and after the compensation are shown in Figure 16. After error compensation, the accuracy of the rotary encoder reaches 0.2°, and it has the same accuracy within the scope of the whole cycle.

## 5. Conclusions and Future Works

The analysis of factors is very important to improve the performance of Hall devices, which provides guidance to the designing and manufacturing process of rotary encoders. In this paper, a model-based rapid diagnosis method for rotary encoders based on Hall plates with a magnetic concentrator is proposed. The parameters of error factors in the possible range are used to fit the error curve by comparing the minimum residual angle error. Meanwhile, the modified PSO algorithm is used to improve the calculation speed.

Using this method, the parameters of the error can be obtained without using high-cost internal measurement. The parameters can reflect the trend of the error factor change and provide an easy way to get error factors after the assembly of the encoder. Using the parameters, small changes during the manufacturing process can be monitored. By keeping the sensor in good working condition, such as a least non-linear distance in different magnet and distance cases, as well as continuous error compensating, this keeps the accuracy in the whole cycle close to an agreement. The method by calculating the results to back-step the presence or absence of error is easier and more effective. Compared to the traditional measuring method, the model-based diagnosis method is a more effective way to locate error factors.

For all of that, the model and error locating method are not perfect; because some factors are sensitive to temperature, while some factors are immune to it. Selective compensation of the parameters can help the temperature characteristics [26,30]. In the future, the models taking into account more factors and the corresponding error, and therefore being more accurate, will be the focus of further work.

## Figures and Tables

**Table 1.** Influence of single factors.

**Factor**	**Calculated Error**	**Impact Factor**
Offset	0.08102	1 mV offset in both axes
Gain mismatch	0.08581	mismatch ratio is 0.003
Phase	0.09999	phase deviation is 0.1°
Non-Linearity	0.07682	magnetic field intensity is 100 mT
Voltage Fluctuation	0.072	1 mV fluctuation in max impact
Calculation Error	0.0078	8 times iterations with the worst case
Other	immeasurable	Other factors
Sum Foreseeable	0.42346	not including immeasurable factors

Note: The calculated error is in degrees. The calculated error is the max value of the situation. As you can see, the sum value is unacceptable. To improve the performance of the sensor, we must control these factors within a rational range.

**Table 2.** Definition and output of parameters.

**Factor**	**Definition of Parameters**				**Output of Parameters**			
	
	**Variable**	**Range (min)**	**Range (max)**	**Step**	**a**	**b**	**c**	**d**
Offset	Vx	−0.01	0.01	0.000001	0.008943	0.005878	0.007500	0.007711
mismatch	Ax	0.99	1.01	0.000001	1.008607	1.000057	0.993573	0.994878
Offset	Vy	−0.01	0.01	0.000001	0.000491	0.007176	−0.006990	−0.003980
mismatch	Ay	0.99	1.01	0.000001	0.994536	0.995089	1.003337	0.992822
Phase	Px	−0.9/pi	0.9/pi	0.00009/pi	0.000368	0.001508	0.003005	0.002122
Linearity	Magx	50	150	1	113	135	71	110

Note: The step and range of parameters are given as the above table. Using the diagnostic method, parameters in different situations can be obtained.
